# The use of singlebeam echo‐sounder depth data to produce demersal fish distribution models that are comparable to models produced using multibeam echo‐sounder depth

**DOI:** 10.1002/ece3.8351

**Published:** 2021-12-09

**Authors:** Marcela Montserrat Landero Figueroa, Miles J. G. Parsons, Benjamin J. Saunders, Ben Radford, Chandra Salgado‐Kent, Iain M. Parnum

**Affiliations:** ^1^ Centre for Marine Science and Technology (CMST) Curtin University Perth WA Australia; ^2^ Australian Institute of Marine Science Nedlands WA Australia; ^3^ School of Molecular and Life Sciences Curtin University Bentley WA Australia; ^4^ Oceans Blueprint Coogee WA Australia; ^5^ Centre for Marine Ecosystems Research School of Science Edith Cowan University Joondalup WA Australia

**Keywords:** bathymetry, demersal fish distribution, habitat model, interpolation, multibeam, singlebeam echo‐sounder

## Abstract

Seafloor characteristics can help in the prediction of fish distribution, which is required for fisheries and conservation management. Despite this, only 5%–10% of the world's seafloor has been mapped at high resolution, as it is a time‐consuming and expensive process. Multibeam echo‐sounders (MBES) can produce high‐resolution bathymetry and a broad swath coverage of the seafloor, but require greater financial and technical resources for operation and data analysis than singlebeam echo‐sounders (SBES). In contrast, SBES provide comparatively limited spatial coverage, as only a single measurement is made from directly under the vessel. Thus, producing a continuous map requires interpolation to fill gaps between transects. This study assesses the performance of demersal fish species distribution models by comparing those derived from interpolated SBES data with full‐coverage MBES distribution models. A Random Forest classifier was used to model the distribution of *Abalistes stellatus*, *Gymnocranius grandoculis*, *Lagocephalus sceleratus*, *Loxodon macrorhinus*, *Pristipomoides multidens*, and *Pristipomoides typus*, with depth and depth derivatives (slope, aspect, standard deviation of depth, terrain ruggedness index, mean curvature, and topographic position index) as explanatory variables. The results indicated that distribution models for *A. stellatus*, *G. grandoculis*, *L. sceleratus*, and *L. macrorhinus* performed poorly for MBES and SBES data with area under the receiver operator curves (AUC) below 0.7. Consequently, the distribution of these species could not be predicted by seafloor characteristics produced from either echo‐sounder type. Distribution models for *P. multidens* and *P. typus* performed well for MBES and the SBES data with an AUC above 0.8. Depth was the most important variable explaining the distribution of *P. multidens* and *P. typus* in both MBES and SBES models. While further research is needed, this study shows that in resource‐limited scenarios, SBES can produce comparable results to MBES for use in demersal fish management and conservation.

## Graphical abstract

This study assesses the performance of demersal fish species distribution models by comparing those derived from interpolated singlebeam (SBES) data with full‐coverage multibeam (MBES) distribution models. The results indicated that the performance of the SBES distribution models was not significantly different from that of the MBES models. While further research is needed, this study shows that in resource‐limited scenarios, SBES can produce comparable results to MBES for use in demersal fish management and conservation.

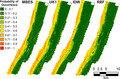

## 1 INTRODUCTION

Seafloor geomorphology has been recognized as an important factor influencing demersal fish distribution both at broad (kilometers) and fine (tens of meters) scales (Demestre et al., 2000; Monk et al., 2011; Moore et al., 2011; Pierdomenico et al., 2015). Hence, various terrain parameters, commonly termed depth derivatives (Garcia‐Alegre et al., 2014), that quantify the geomorphology of the seafloor (e.g., slope, aspect, curvature, and rugosity) have been included in distribution models of demersal fish (Becker et al., 2009; Demestre et al., 2000; Ierodiaconou et al., 2011; Lucieer & Pederson, 2008; Young & Carr, 2015; Young et al., 2010).

Broad‐scale depth derivatives can help to explain the distribution of species with a preference for large‐scale features (Wilson et al., 2007). However, the fine‐scale associations within the landscape context are also important in structuring demersal species distribution (Anderson et al., 2009). Consideration of habitat associations at different scales is therefore recommended when modeling habitat availability for species (Anderson et al., 2009; Garcia‐Alegre et al., 2014; Jones & Brewer, 2012; Monk et al., 2011; Pittman & Brown, 2011).

Despite the importance of the seafloor geomorphology in determining habitat for fisheries and conservation management applications, only 5%–10% of the world's seafloor has been mapped with multibeam echo‐sounders (MBES; Sandwell et al., 2003; Wright & Heyman, 2008). This low percentage is because accurate characterization is usually time‐consuming, expensive, and technically challenging. MBES collect high‐resolution bathymetric information, cover a wide swath area, and usually acquire an almost continuous coverage of the study area. However, they typically require greater financial and technical resources for operation and data analysis than the simpler and more cost‐effective singlebeam echo‐sounders (SBES). In contrast to MBES, SBES provide limited coverage, ensonifying only a small area directly below the echo‐sounder. Interpolation between SBES transects is therefore required to fill the gaps to produce a continuous seafloor map. If useful seafloor maps could be produced from SBES interpolated data, the cost to produce accurate demersal fish distribution models for conservation and management would be significantly reduced.

While there are numerous interpolation methods available to produce continuous bathymetry data from sparse datasets, no consensus has been reached on which method is the most accurate (Bello‐Pineda & Hernández‐Stefanoni, 2007; Curtarelli et al., 2015), yet useful in species distribution modeling. For other applications, like navigation, protocols and requirements are well established to fulfil legal requirements of scale and accuracy (Mills, 2015).

The performance of different interpolation methods depends upon the seabed characteristics, and sampling density and distribution (Arun, 2013; Erdogan, 2009; Moskalik et al., 2013). In this study, the accuracy of three commonly used methods proven to model bathymetry effectively were compared, including inverse distance weighting (IDW), radial basis function (RBF), and Kriging (Moskalik et al., 2013; Sanchez‐Carnero et al., 2012). Kriging also included testing three variations; ordinary (OK) and universal with a first‐ and second‐degree detrending (UK1 and UK2).

The overall aim of this study was to test the ability of cost‐effective methods (i.e., SBES‐derived depth data) for modeling demersal fish species distributions. This study compared the accuracy of demersal fish species distribution models produced using SBES bathymetry and depth derivatives at different scales with those derived from MBES data. Specifically, the following were tested: (1) the accuracy of the three common interpolation methods (IDW, RBF, and Kriging) in producing continuous bathymetry using SBES data; (2) the similarity between the resulting interpolated SBES bathymetries and depth derivatives with the MBES bathymetry and depth derivatives; and (3) accuracy of demersal fish distribution models (Random Forest [RF]) constructed using SBES and MBES bathymetry and depth derivatives at different scales.

## 2 METHODS

### 2.1 Study area and data collection

The Ningaloo Reef is located off the coast of northwest Australia. It is the longest fringing coral reef in Australia and is recognized as a global biodiversity hotspot, home to a wide variety of wildlife, including many endangered species (Gazzani & Marinova, 2007; Schonberg & Fromont, 2012). Between 2006 and 2009, a multi‐institutional research program was conducted in the Ningaloo Marine Park (NMP) by Western Australia Marine Science Institute partners (Waples & Hollander, 2008) and included the collection of SBES data (Colquhoun et al., 2007). The assemblage and relative abundance of demersal fishes were surveyed using Baited Remote Underwater Stereo‐Video Systems (stereo‐BRUVS) between March 26 and May 6, 2009 (Colquhoun et al., 2007), following procedures described in Harvey et al. (2007). The resulting presence and absence records of different fishes at each stereo‐BRUVS site were used in this study.

**FIGURE 1:**
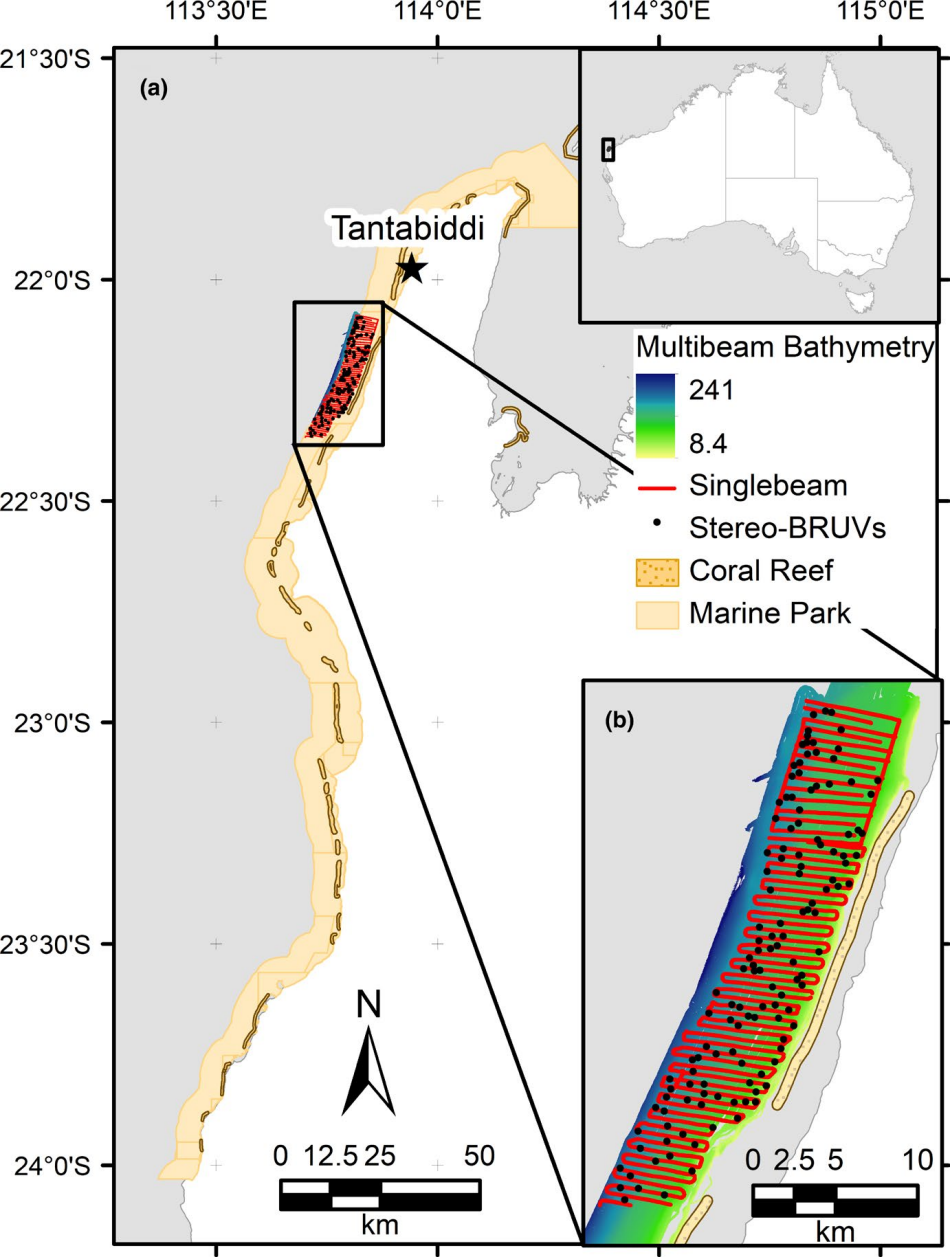
Map indicating (a) the study site located in the north section of the Ningaloo Marine Park, and (b) the locations of single‐beam echo‐sounder survey tracks shown in red and stereo BRUVS deployment sites shown with black dots

Multibeam echo‐sounders data were collected in particular areas of the NMP in 2008, by Geoscience Australia (GA; Brooke et al., 2009). SBES bathymetric data were collected with a Simrad EQ60 echo‐sounder (38 and 200 kHz frequencies), mounted on the *RV Cape Ferguson* and *RV Solander* vessels for the 2006 and 2008 surveys, respectively, using 500‐m transect spacing in both surveys (Colquhoun et al., 2007). An area of the NMP located 2.5 km from the mainland coast to 7.5 km offshore, where SBES, MBES, and stereo‐BRUVS data overlapped, was selected as the study site (Figure 1). This area extended approximately 35 km parallel to the coast, with seafloor depths ranging from approximately 20 to 130 m. The MBES bathymetric data covering the area of interest for this study were downloaded as a 3‐m resolution grid from the GA website (Figure 1).

### 2.2 SBES data processing

Depth values from the SBES data were extracted by first using the readEKraw MATLAB toolkit to read the Simrad raw data into MATLAB (Rick Towler, NOAA Alaska Fisheries Science Centre, Seattle, WA, USA). Then, depth was estimated from the differential of a mean waveform created from 10 consecutive pings, to improve the signal‐to‐noise ratio. After averaging, the mean distance between depth estimates was 40 m. Depth values were corrected for tidal height using the predicted values of tide for Tantabiddi (Department of Transport, 2006) and adjusted to be relative to the Australian Height Datum (0.30 m above Chart datum). Erroneous depth values (either positive or clearly unrealistic in relation to surrounding values) were removed manually.

After filtering and averaging, 11,122 SBES depth records were included in the analysis and ranged from 18 to 127 m, with a mean depth of 77.26 m. The coefficient of skewness was below the threshold of ±1 (0.14), indicating the data had a symmetrical normal distribution; thus, no transformation was required for geostatistical analysis (Kerry & Oliver, 2007). While the distance between transects was 500 m, the resolution of the final interpolated surface was selected based on the distance between soundings along track, in an attempt to keep the higher resolution possible in the axis with higher density of data. The number of soundings included in the calculation of each pixel of the final surface was based on the number of neighbors included in the analysis and the radius of search.

### 2.3 Spatial interpolation methods

Interpolations of depth data were conducted in the R software package (R Development Core Team, 2017) using IDW, RBFs, and Kriging, following methods detailed in Mitas and Mitasova (1999). During IDW analyses, multiple powers (ranging from 0.5 to 6, in 0.5 increments) and selected neighborhoods (50, 100, and 150 raster's cells) were tested using the gstat package (Pebesma, 2004). As RBF estimation in unsampled areas can be dependent on one of five possible base functions (Buhmann, 2003), this study used completely regularized spline (CRS) and multiquadratic (M) RBFs, which have been found to be accurate in the production of depth interpolation (Erdogan, 2009). The RBF functions use smoothness and robustness parameters to control the level of smoothness and stability of the interpolation. These two parameters were optimized in the geospt package (Melo et al., 2012), which uses cross‐validation to minimize root‐mean‐square errors (RMSE). The performance of CRS and M with neighbors set at 50, 100, and 150 raster cells was tested. During Kriging, a semi‐variogram was created to test spatial autocorrelation and a Gaussian model fitted to the sampling points (Cressie, 1993). Isotropic and anisotropic variograms for ordinary and universal Kriging with a first‐ and second‐degree detrending with 100 and 150 neighborhoods were tested using the gstat package (Pebesma, 2004).

### 2.4 Selection and comparison of best interpolation approaches

For each scenario of IDW and RBF, the best method was the one that produced the lowest RMSE, using leave‐one‐out cross‐validation (Hengl, 2009). In Kriging, the scenario with the lowest absolute difference between the RMSE and average Kriging standard error (ASE; Asa et al., 2012) was chosen as the best model.

The interpolated surfaces from the best performing IDW, RBF, and Kriging methods selected were compared with the MBES surface using correlation and regression analyses to assess the relationship between the overall surfaces (interpolated vs. MBES). Correlation and regression analyses were undertaken for subsets of raster pixel values corresponding to four different buffer distances (intervals) perpendicular to the original SBES track (0–100, 101–200, 201–300, and 301–400 m). Pixel values of the interpolated SBES surface contained in each interval were compared with the MBES pixel of the same interval using correlation and regression analyses. Values closer to the original SBES data (i.e., within the 0–100 m interval) were expected to be accurate regardless of interpolation techniques, while the accuracy of values further away (301–400 m interval) was expected to depend upon the interpolation technique. The significance of the correlation coefficient *r* and the coefficient of determination *R^2^* was also tested.

### 2.5 Digital elevation models

A digital elevation model (DEM) in which each pixel corresponds to the interpolated values was produced using the interpolation method with the lowest RMSE. Additional DEMs were produced at resolutions of 9, 15, and 25 m. To achieve this, a Gaussian filter (5 × 5 kernel) was applied to the interpolated bathymetry to reduce the effect of noise that can be particularly problematic at the edges of overlapping transects (Stephens & Diesing, 2014). The DEM was then resampled at the corresponding resolutions using a bilinear method with the raster package (Hijmans, 2016). DEMs were also produced from MBES data, which were filtered in the same way as SBES data.

### 2.6 Seafloor depth and its derivatives

Depth derivatives including slope, aspect, terrain ruggedness index (TRI), standard deviation of depth (SD), topographic position index (TPI), roughness, and mean curvature (MNC) were calculated using a 3 × 3 window analysis at four different resolutions (Table 1). The finest scale of analysis was fixed by the resolution of the MBES data (3 m), while the other three were chosen based on the spatial dependence of the species distribution. A variogram analysis was used to identify the maximum distance at which the species present spatial dependency, which is called the range. The scales were chosen to cover the span between the 3‐m resolution and the range of the species (>4 km) (Holmes et al., 2008). Therefore, the resolutions were set at 3, 9, 15, and 25 m. Depth derivatives were produced for SBES and MBES DEMs at the four resolutions.

**TABLE 1:** Depth derivatives produced from SBES depth data

Variable	Abbreviation	Software	Reference
Slope	Slope	R (raster)	Horn (1981)
Aspect			
Northness	NS	R (raster)	Horn (1981)
Eastness	WE	R (raster)	Horn (1981)
Standard deviation of depth	SD	R	Lecours et al. (2016)
Terrain ruggedness index	TRI	R (raster)	Wilson et al. (2007)
Topographic position Index	TPI	R (raster)	Wilson et al. (2007)
Roughness	Roughness	R (raster)	Wilson et al. (2007)
Mean curvature	MNC	Landserf v 2.3	Wood (1996)

Abbreviation: SBES, singlebeam echo‐sounders.

Slope and aspect were calculated in a raster package (Hijmans, 2016), following methods described by Horn (1981). Aspect was split into two variables using trigonometric functions: northness (NS) and eastness (WE), where NS is the cosine of aspect and varies from −1 (south) to 1 (north), and WE is the sine of aspect varying from −1 (west) to 1 (east), as per Deng et al. (2007).

### 2.7 Demersal fish species distribution models

Random Forest, as proposed by Breiman (2001), was used in this study to model the distribution of demersal species of fish. RF is a machine‐learning technique that has been shown to outperform conventional statistical techniques, such as linear and generalized additive regression models when used to model the distribution and diversity of demersal fish (Knudby et al., 2010; Smolinski & Radtke, 2017). RF classifier algorithms (Breiman, 2001) were used to model the distributions of Starry Triggerfish (*Abalistes stellatus*), Robinson's Seabream (*Gymnocranius grandoculis*), Silver Toadfish (*Lagocephalus sceleratus*), Sliteye Shark (*Loxodon macrorhinus*), Goldband Snapper (*Pristipomoides multidens*), and the Sharptooth Snapper (*Pristipomoides typus*), using seafloor depth and its derivatives from SBES and MBES. While fishes of high commercial value were prioritized, the low number of presences of these species in stereo‐BRUVS in the study area inhibited the production of accurate models. Consequently, species chosen for analyses were not fisheries target species, with the exception of *P. multidens*, *P. typus* that are target species in Western Australian commercial and recreational fisheries (Marriott et al., 2012). The six species distribution models were constructed using presence/absence records from stereo‐BRUVS data. These species are all considered reef‐associated, with a habitat generalist's relatively broad distribution (Fitzpatrick et al., 2012; Kalogirou, 2013; Randall, 1967), and displayed ≥35 presences in BRUVS data.

Depth derivatives included in the species distribution model are listed in Table 1. Four RF (43) models were created for each of the six species using MBES data and SBES, including depth and its derivatives (24 models in total) using the R (R Development Core Team, 2017) package randomForest (Liaw & Wiener, 2002). The performance of each model was evaluated using the area under the receiver operator curve (AUC), which summarizes the sensitivity and specificity of the model (Manel et al., 2001). Seventy percent of the data was used to train the RF, and the rest to test the accuracy of predictions from the model. The trained RF was used to predict the probability of presence of the species in the 30% reserved for testing and residuals then calculated (Zhang et al., 2020). The accuracy of the models was tested using a five‐fold cross‐validation procedure, and significance of the difference in mean AUC between the interpolated SBES and the MBES models was examined using a *T*‐test. Partial dependence plots were used to explore the relationship between the terrain variables and the presence of the species (Friedman, 2001).

## 3 RESULTS

### 3.1 Selection and comparison of best interpolation approaches

#### 3.1.1 Inverse distance weighting

The best IDW model produced a power parameter of three and a RMSE of 0.39, although similar RMSEs were observed for 3 and 3.5 powers (Supporting Information). Lower RMSEs were found when the number of neighbors was decreased for powers between 2 and 3.

#### 3.1.2 Radial basis function

The best RBF model had a multiquadratic function (M) with a RMSE of 0.39. CRS had a greater associated error above 0.45. No differences were observed when the number of neighbors was increased (Supporting Information).

#### 3.1.3 Kriging

Initial variogram analysis showed presence of spatial structure in the data indicating it was suitable for geostatistical analysis. An anisotropy was found in the data with a major axis parallel to the coast where less variation was observed and a minor axis perpendicular to the coast in which much more rapid changes in depth occurred. The anisotropy persisted after a first‐ (UK1) and second‐degree (UK2) detrending. When fitting a theoretical model to the empirical variograms, Gaussian variograms displayed the best fit. The distance at which the spatial autocorrelation reached the sill (called the range) was between 428 and 720 m (Supporting Information).

The best fit Kriging interpolation was the universal Kriging (ASE‐RMSE and RMSE of 0.034 and 0.332, respectively) with UK1 detrending, using anisotropic variograms. In most cases, interpolation using anisotropic variograms had lower RMSEs than those using isotropic variograms. Ordinary and universal Kriging with UK1 detrending performed similarly, with low values of RMSE. For OK and UK1, higher ASEs than RMSEs were estimated when an anisotropic variogram was used, indicating an underestimation of the variability. Higher RMSEs than ASEs were observed for OK and UK1 when an isotropic variogram was used (Supporting Information), indicating an overestimate of the variability. Universal Kriging with UK2 detrending with an isotropic and anisotropic variogram also overestimated the variability. A slightly lower value of ASE‐RMSE was observed for OK and UK1, when an anisotropic variogram was used. Universal Kriging with UK2 detrending had, in general, the worst performance, with higher values of RMSE and greater difference between ASE and RMSE. Kriging produced the lowest RMSE (0.332) and therefore best performance of the three interploation techniques compared with IDW and RBF (0.398 and 0.397, respectively).

**FIGURE 2:**
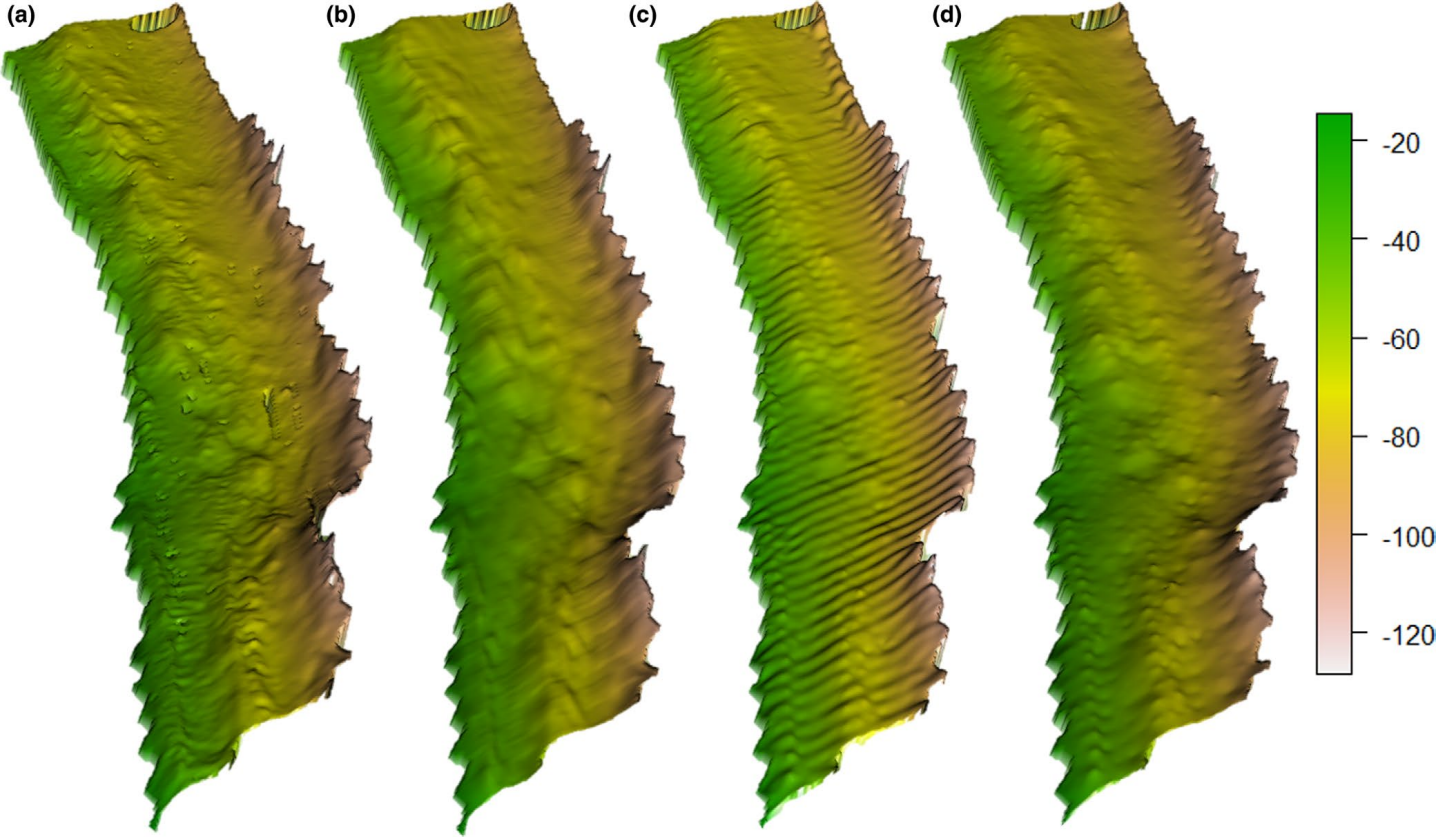
Sun‐illuminated bathymetry of the study site using a 3D projection for (a) MBES and the best SBES data interpolations in this study using: (b) universal Kriging with first degree of detrending, (c) inverse distance weighting, and (d) radial basis function

When comparing the SBES interpolated surfaces with the gridded depth surface from the MBES data, a good correlation (all coefficients of determination were >0.99) was found between the MBES data and the three SBES interpolated DEMs. Significant linear relationships between the MBES data and the interpolated data were found for all methods (*p* < .001). A slight decrease in the coefficient of determination (*R* ^2^) was observed when the distance from the original SBES track was increased (Supporting Information). UK1 had the highest *R* ^2^ for all intervals of distance, closely followed by RBF. IDW had the lowest values of R^2^ for all the distances and particularly for the areas further away from the SBES data (400 m with a *R* ^2^ decrease from 0.997 to 0.990). The MBES and SBES interpolated surfaces are comparable with some visible artefacts, particularly for the IDW and RBF surfaces (Figure 2).

### 3.2 Seafloor depth and its derivatives

In general, a larger variation was found in the depth derivatives, based on the MBES data compared with the SBES interpolated data; this was particularly true for the derivatives based on the highest resolution bathymetry (3 m, Supporting Information). The derivatives based on the interpolated SBES data had similar means and SDs to the MBES derivatives at a broader scale (25‐m resolution).

A gentle slope (≈1°) was found in the study area directed predominantly oriented north‐west, shown by the predominately positive NS and negative WE values, with high variation observed at the 3‐m resolution, particularly for the MBES data. In all cases, the MNC had slightly negative values associated with concave areas in the terrain, although both positive and negative MNCs were observed. The SD of depth, TRI, TPI, and roughness presented a mean close to zero at the highest resolution, indicating low terrain variability at fine scale. Higher means and SDs were observed for the MBES data, and the interpolated surfaces, as resolution decreased (i.e., the cell size increased).

**FIGURE 3:**
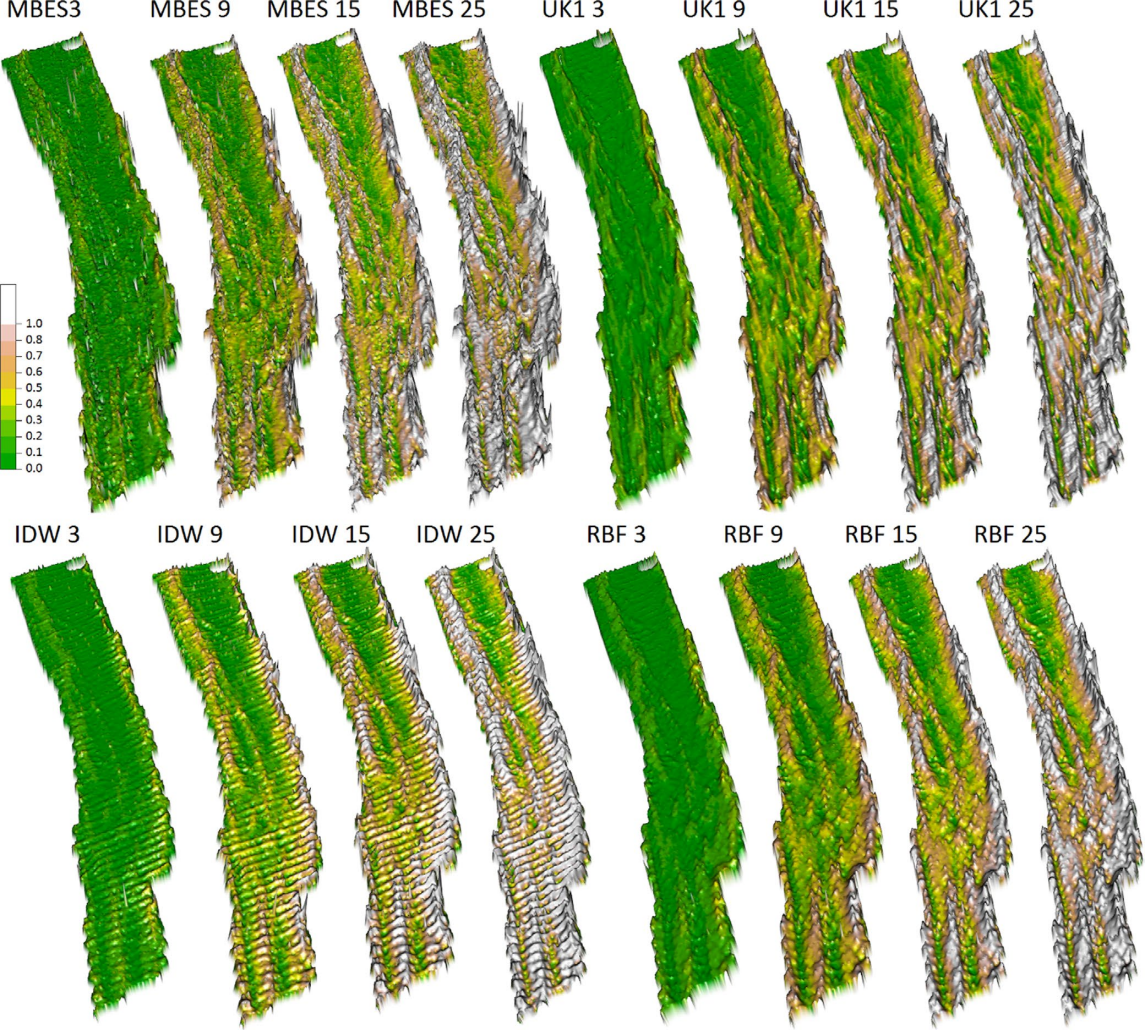
Sun‐illuminated 3D projection of the roughness derivate from the MBES and interpolated SBES data using universal Kriging with a first‐degree detrending (UK1), inverse distance weighting (IDW), and radial basis function (RBF). The four resolutions included in the analysis are shown

Derivatives based on the interpolated bathymetries presented different levels of artefacts associated with inaccuracies in the interpolation process. Pronounced artefacts were observed in all derivatives, particularly those based on the IDW interpolated bathymetry (see Figure 3, for example maps of derived roughness).

### 3.3 Demersal fish species distribution models

Demersal fish distribution modeling performance was species‐dependent. The distribution of *A. stellatus*, *G. grandoculis*, *L. sceleratus*, and *L. macrorhinus* was poorly modeled by the MBES and interpolated SBES data (Supporting Information) with mean AUCs below 0.7 (Hosmer et al., 2013). The distribution of *P. multidens* and *P. typus* was well modeled using the variables included in the analysis, with AUCs above 0.8 for both the MBES and SBES interpolated models. No significant differences were observed between the mean AUCs of the models produced using MBES data compared with the SBES models (*p* < .05).

### 3.4 Variables importance

Even though accuracy of the models for *A. stellatus*, *G. grandoculis*, *L. sceleratus*, and *L. macrorhinus* was below the acceptable level, the analysis of the variables' importance can provide insight of the factors affecting their distribution. For *G. grandoculis* and *L. sceleratus*, depth was the most important variable in the MBES and SBES models (Supporting Information). TRI and MNC at a fine to medium scale were also important in the *L. sceleratus* MBES model; yet, in the SBES models, these variables had only a marginal contribution. Variables related to terrain variability (e.g., SD and TRI) were important in the *A. stellatus* model, at both broad and fine scales, for both the MBES and SBES models. The slope orientation in both the northness and eastness components was also important in the MBES model but at specific scales of analysis, with eastness being more important at the finest scale (3‐m resolution), while northness was relevant at medium to large scales (9–25‐m resolution). For the *L. macrorhinus* model, depth had slightly higher importance followed by roughness, SD, TRI, and slope, at both fine and broad resolutions for the MBES and the SBES UK1 model. Mean curvature was important at a broad scale (25 m), while northness was relevant at a fine scale (3–9 m).

Depth was the most critical variable in modeling the distribution of both *P. multidens* and *P. typus* for the MBES data, and the models based on the interpolated SBES data (Supporting Information). For *P. multidens*, roughness, slope, SD, and TRI, followed depth in importance at both fine and broad scales; MNC had also a significant contribution but only at a medium scale (15 m). In the *P. typus* model, the eastness component of slope orientation was important at a fine scale (3 m), with the rest of the variables having a lower contribution in the MBES model. For the interpolated SBES models, no clear pattern was observed with variables having similar levels of contribution at a fine or broad scale.

### 3.5 Probability of occurrence of *P. typus*

**FIGURE 4:**
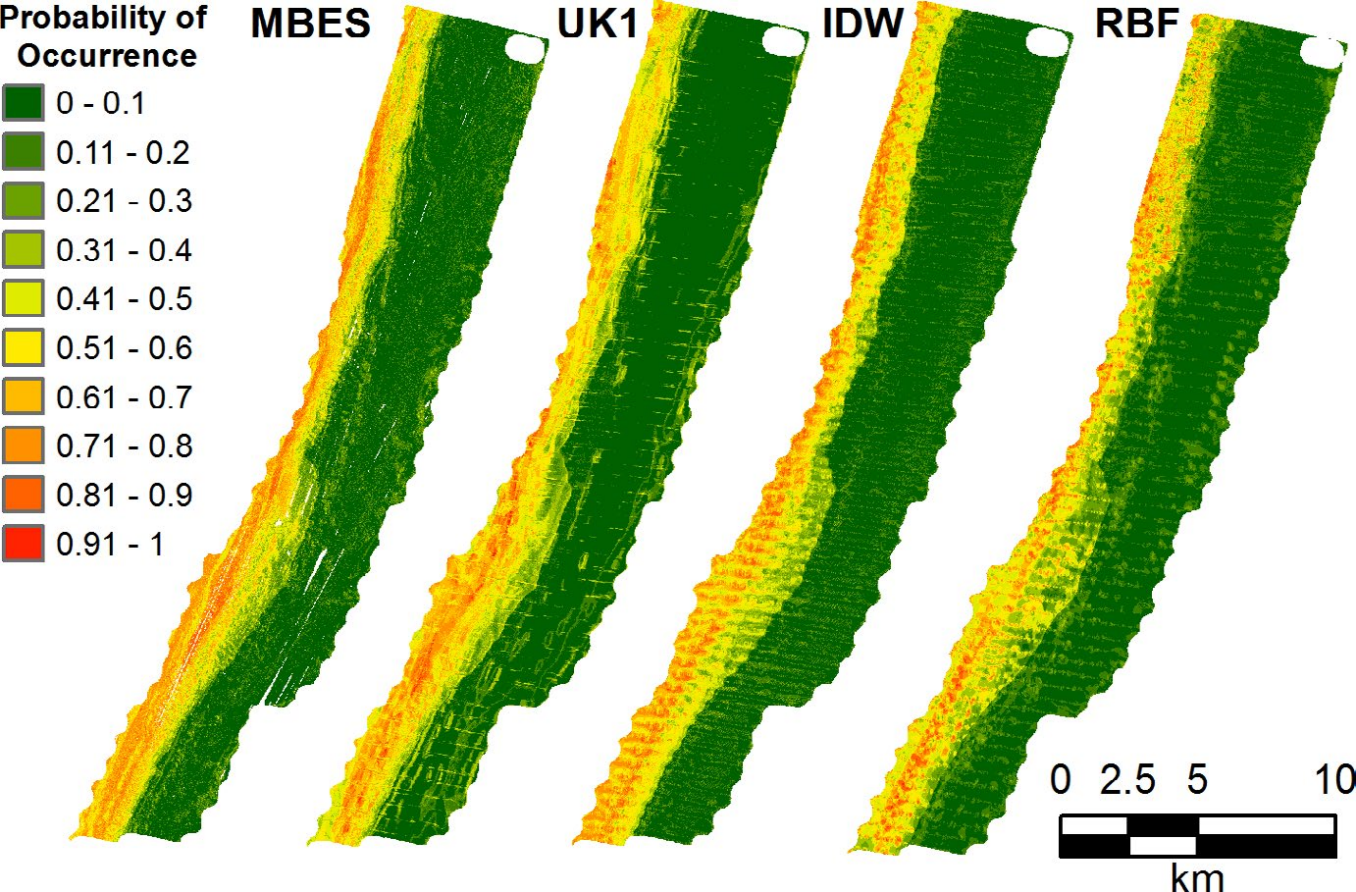
Maps of probability of occurrence of *Pristipomoides typus* based on depth and depth derivatives of the MBES and the three interpolation techniques tested: Universal Kriging with first degree of detrending (UK1), inverse distance weighting (IDW) and radial basis function (RBF)

**FIGURE 5:**
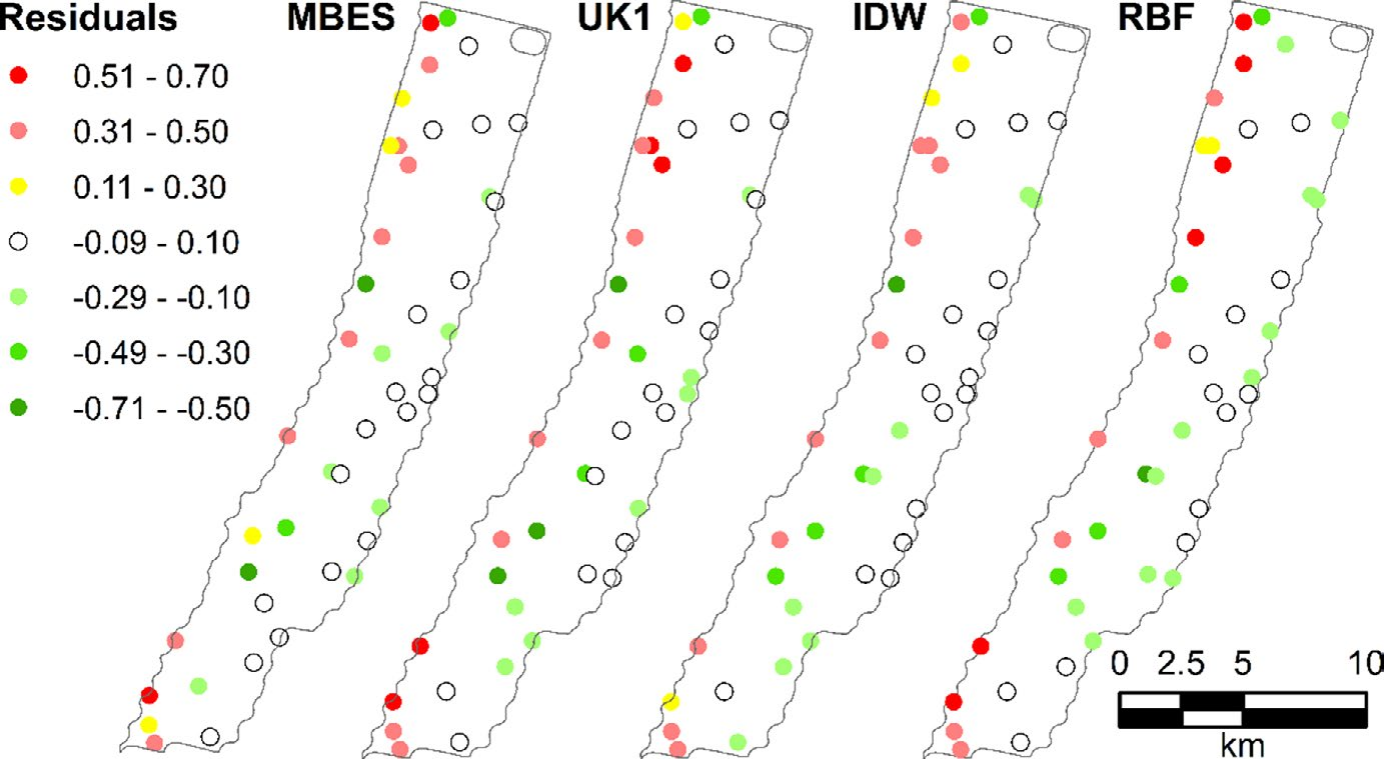
Spatial distribution of the residuals of the Random Forest predicting the testing portion of the *Pristipomoides typus* data. Positive values corresponds to under predictions while negative values represent over predictions

*Pristipomoides multidens and P. typus* have very similar habitat distributions, with a preference for deeper areas (Parrish, 1987b; Sih et al., 2017). The probability of occurrence of *P. typus* showed higher probabilities of occurrence in deeper areas and lower in the rest of the study area for both MBES and SBES models (Figure 4, *P. typus* shown only, due to similarities of distribution maps of the two species). Similar spatial patterns were observed for all the models; however, the SBES models presented visually recognizable artefacts in the probability of occurrence derived from errors in the interpolations (Figure 4). In particular, artefacts in the SBES RBF model were more evident in the areas of high probability of occurrence. Spatial clustering of residuals was observed in all models of *P. typus*, including the MBES model, with underprediction in the deeper areas and overprediction in the shallows observed (Figure 5). Overprediction in the shallower areas was less pronunced in the MBES model compared with the interpolated models.

## 4 DISCUSSION

### 4.1 Selection and comparison of best interpolation approaches

In this study, universal Kriging with a first‐order detrending (UK1) was found to be the method of choice to interpolate the SBES depth data, over IDW and RBF. This was based on the surface produced by UK1 having the lowest RMSE in the leave‐one‐out cross‐validation test and the highest correlation with the MBES data. Similar results of Kriging outperforming IDW and RBF have been reported before when modeling elevation data (Arun, 2013; Bello‐Pineda & Hernández‐Stefanoni, 2007; Curtarelli et al., 2015; Moskalik et al., 2013; Zimmermann & Kienast, 1999). The better performance of Kriging in this study could have been related to the sampling design, as geostatistical methods are best suited for modeling irregularly distributed data (Curtarelli et al., 2015). The data analysed here were not equally spaced, as there was a high density of data in the transects but also significant areas without any data between the transects. One of the disadvantages of Kriging is that some knowledge of geostatistics is needed to produce the best possible result. For instance, an exploration of the (variogram) model needs to be carried out to determine which theoretical variogram should be used and whether a detrending process or the use of an anisotropic variogram is required. To assist this process, there is well‐established software and guidance available to carry these steps out (Glenn et al., 2016).

### 4.2 Seafloor depth and its derivatives

Sampling areas with sparse data in the form of SBES lines produce track line artefacts when interpolated (Hell & Jakobsson, 2011). These artefacts affect the depth derivatives that reflect not only real variations in the DEM but also false variations. In this study, all the interpolation methods produced artefacts, which affected the depth derivatives. Hell and Jakobsson (2011) proposed gridding with minimum curvature splines in tension at multiple grid resolutions overcame this issue by using a high‐resolution grid in the areas with high volumes of data and lower resolution in no data areas. This method could reduce the artefacts produced in this study, although a practical limitation of the Hell and Jakobsson (2011) approach is the large computational requirements. Changes in resolution have different effects in the depth derivatives, related to the specific terrain being studied (Deng et al., 2007). Slope, for example, has the general pattern of a decrease as the resolution decreases (Wilson et al., 2007). This general pattern was observed in this study, with a significant reduction in the variability of the slope at the lowest resolution of analysis. The opposite was observed for the derivatives measuring terrain variability (TRI, roughness, and SD), with an increase of variability for the lower resolutions (Friedman et al., 2012). The inclusion of different resolutions of derivatives can increase the possibilities of having relevant information at the correct scale for the species under study. However, the fine‐resolution derivatives based on the SBES data failed to capture the fine‐scale variability observed in the high‐resolution MBES derivatives. Therefore, species whose distribution is influenced by terrain variability at a fine scale are less likely to be well modeled by SBES interpolated data.

### 4.3 Demersal fish species distribution models

The performance of the distribution models was species‐dependent, but no significant difference was observed between the accuracy of the models constructed using MBES and SBES data. The species included in this study are demersal carnivores with a certain degree of generalist and/or opportunistic feeding behavior (Carpenter & Niem, 1998; Gutteridge et al., 2011; Randall, 1967; Rousou et al., 2014). Four of them including *G. grandoculis*, *L. macrorhinus*, *P. multidens*, and *P. typus* belong to families that have been found in a variety of benthic habitats and classified as habitat generalist with relatively broad cross‐shelf distribution in a previous study in the NMP (Fitzpatrick et al., 2012). However, the difference between habitat‐generalist and habitat‐specialist species is related to the frequency of occurrence of the species in the study area (Jarnevich et al., 2015). In the present study, three species including *A. stellatus*, *G. grandoculis*, and *L. sceleratus* had a generalist behavior with high prevalence in the sampling points (>40%); the models of these species had poor performance for both MBES data and SBES data. Previous studies, have found that generalist species are harder to model, while specialist species are usually better modeled using environmental variables (Franklin et al., 2009). In this study, this pattern was found to be particularly true in the two extremes of the prevalence scale with *A. stellatus* having the highest prevalence (>70%) and its models having the lowest accuracy (AUCs <0.5), while *P. multidens* had a low prevalence (<30%) and had the highest accuracy (AUCs >0.9). The generalist behavior of *A. stellatus*, *G. grandoculis*, and *L. sceleratus* might be due to the extent and the temporal resolution of the study; for example, some species might use specific feeding habitats at night while using different habitats during the day (Harvey et al., 2012). *Loxodon macrorhinus*, on the other hand, had the lowest prevalence in the study site, but its distribution was poorly modeled by depth and its derivatives; a possible explanation for these results can be that water column variables rather than not terrain variables are more closely related to its distribution. A previous study by Gutteridge et al. (2011) found that *L. macrorhinus* prefers areas with clear water when compared with other areas with less water clarity; therefore, the inclusion of water column variables could improve the performance of the models for this species.

The RF models showed that both *P. multidens* and *P. typus* prefer deep waters with some level of bottom complexity. These results are in accordance with previous studies that showed *P. multidens* is a schooling deeper‐water (40–245 m) demersal species found in rocky reefs, coral reef areas, and loose rock/pebble/gravel areas close to steep drop‐offs (Allen, 1985).

For *P. typus*, the preference of deeper areas has been supported by other studies, which indicate a preference for non‐flat seafloors (Parrish, 1987a) and specific depth ranges (Fry et al., 2006). Fry et al. (2006) found a preference of *P. typus* for deeper areas with more fish caught in depth ranges between 125 and 150 m. In a more recent study on the Great Barrier Reef, a series of stereo‐BRUVS were deployed along the shelf‐edge and found *P. typus* was only present in sampling stations between 115 and 250 m (Sih et al., 2017). The high importance of depth to explain *P. typus* distribution may not be the primary factor driving its distribution, per se. Depth is a variable correlated with a combination of biotic and abiotic environmental conditions that might be more related to the distribution of *P. typus* (Sih et al., 2017). The preference of *P. typus* for deep and non‐flat areas was identified by the MBES model and was captured by the model based on the interpolated DEM. For the MBES model and the interpolated model, the medium and broader scale variables had higher importance in the construction of the models. There was a general trend across models for an association between the presence of *P. typus* and areas with increased complexity. The final prediction of the probability of occurrence for *P. multidens* and *P. typus*, based on the MBES and interpolated models, was similar. However, under‐ and overestimation of probability of occurrence were present in all the models, while spatial clustering of the residuals was more evident in the RBF interpolated model.

## 5 CONCLUSION

Interpolated SBES depth data can be used to provide useful species distribution models for broad‐scale habitat associated specialists, when compared with MBES models, with the highest performing SBES model derived using Kriging. Thus, while MBES data should be collected where possible, surveys that are financially restricted may benefit from the less labor‐ and cost‐intensive option of SBES. The possibility of producing models with comparable accuracy to the MBES data can be particularly useful for shallow turbid areas where satellite derivative bathymetry is not suitable and the use of MBES offers little advantage because of its narrow coverage. This is not without caveats, however, as SBES interpolated models are expected to perform poorly for species affected by fine‐scale variation of the terrain, because of its failure to capture fine‐scale variation of the terrain complexity. Models based on interpolated SBES data can produce accurate models for species strongly influenced, directly or indirectly, by depth. Further studies including a wide range of species and terrains with different levels of complexity are needed to confirm the findings of the present study. Different species with specific levels of habitat specialization and relationship with the environmental variables might respond differently. The inclusion of other variables like seafloor backscatter, as a descriptor of substrate type, may help to increase the accuracy of the models for some species.

## CONFLICT OF INTEREST

No authors have any conflicts of interest to report.

## AUTHOR CONTRIBUTIONS

**Marcela Montserrat Landero Figueroa:** Conceptualization (equal); Formal analysis (equal); Investigation (equal); Visualization (equal); Writing‐original draft (equal); Writing‐review & editing (equal). **Miles J. G. Parsons:** Conceptualization (equal); Methodology (equal); Supervision (equal); Writing‐original draft (equal); Writing‐review & editing (equal). **Benjamin J. Saunders:** Conceptualization (equal); Formal analysis (equal); Methodology (equal); Supervision (equal); Writing‐review & editing (equal). **Ben Radford:** Formal analysis (equal); Methodology (equal); Writing‐review & editing (equal). **Chandra Salgado‐Kent:** Conceptualization (equal); Writing‐original draft (equal); Writing‐review & editing (equal). **Iain M. Parnum:** Conceptualization (equal); Investigation (equal); Supervision (equal); Writing‐original draft (equal); Writing‐review & editing (equal).

## Supporting information

Supplementary Material

## Data Availability

Singlebeam echosounder data can be found at: https://osf.io/g7sq4. Stereo‐BRUVs data can be obtained by request to Saunders, B (ben.saunders@curtin.edu.au). Multibeam data can be downloaded directly from the GeoScience Australia website: https://services.ga.gov.au/site_3/rest/services/Marine_Survey_Multibeam_Bathymetry/MapServer/30.
