# Supplementary material for: The use of singlebeam echo‐sounder depth data to produce demersal fish distribution models that are comparable to models produced using multibeam echo‐sounder depth
Source: Ecol Evol. 2021 Dec 9;11(24):17873–84. doi: 10.1002/ece3.8351 (PMC8717343; doi:10.1002/ece3.8351)
Supplement: Supplementary file 1 — Supplementary Material [file ECE3-11-17873-s001.docx]

**Supplementary information**

Table S1. Root mean squared error (RMSE) for the different combinations of powers and number of neighbours tested in the inverse distance weighting interpolation. The best interpolation is indicated by the lowest value of RMSE.

| Power | RMSE | | |
| --- | --- | --- | --- |
|  | Neighbours | | |
|  | 50 | 100 | 150 |
| 2 | 0.477 | 0.559 | 0.621 |
| 2.5 | 0.410 | 0.425 | 0.434 |
| 3 | 0.398 | 0.400 | 0.401 |
| 3.5 | 0.398 | 0.398 | 0.398 |
| 4 | 0.401 | 0.401 | 0.401 |
| 4.5 | 0.404 | 0.404 | 0.404 |
| 5 | 0.406 | 0.406 | 0.406 |
| 5.5 | 0.409 | 0.409 | 0.409 |
| 6 | 0.411 | 0.411 | 0.411 |

Table S2. Root mean squared error (RMSE) for the multiquadratic (M) and completely regularized spline (CRS) radial basis functions (RBFs). Three scenarios of a maximum number of neighbours (n) are shown, and the lowest value of RMSE is shown in bold.

| Type | RMSE | | |
| --- | --- | --- | --- |
|  | Neighbours | | |
|  | 50 | 100 | 150 |
| M | **0.397** | **0.397** | **0.397** |
| CRS | 0.456 | 0.459 | 0.460 |

Table S3. Parameters of the Gaussian models fitted to the empirical variograms comparing Ordinary, and Universal kriging first and second degree detrending, as well as isotropic and anisotropic directionality. Partial sill (psill) is the sill minus the nugget effect.

| Type | Directionality | psill | range |
| --- | --- | --- | --- |
| Ordinary Kriging | Isotropic | 28 | 617 |
|  | Anisotropic | 24 | 720 |
| Universal Kriging 1 | Isotropic | 8 | 464 |
|  | Anisotropic | 7 | 519 |
| Universal Kriging 2 | Isotropic | 6 | 428 |
|  | Anisotropic | 6 | 492 |

Table S4. Root mean square error (RMSE) from cross-validation for the kriging interpolations. The model with the smallest difference between the average kriging standard error (ASE) and RMSE is marked in bold.

| Type | Directionality | Neighbours | | | | | |
| --- | --- | --- | --- | --- | --- | --- | --- |
|  |  | 100 | | | 150 | | |
|  |  | RMSE | ASE | ASE-RMSE | RMSE | ASE | ASE-RMSE |
| Ordinary Kriging | Isotropic | 0.418 | 0.360 | -0.058 | 0.420 | 0.359 | -0.061 |
|  | Anisotropic | 0.342 | 0.381 | 0.039 | 0.343 | 0.381 | 0.038 |
| Universal Kriging 1 | Isotropic | 0.387 | 0.342 | -0.045 | 0.388 | 0.342 | -0.046 |
|  | Anisotropic | 0.331 | 0.366 | 0.034 | 0.332 | 0.366 | **0.034** |
| Universal Kriging 2 | Isotropic | 4.604 | 0.341 | -4.263 | 19.802 | 0.341 | -19.461 |
|  | Anisotropic | 3.151 | 0.366 | -2.785 | 3.645 | 0.366 | -3.279 |

Table S5. Performance of the models measured by the five-fold cross-validation mean AUCs. The results for the species modelled are shown for MBES and interpolated SBES data using universal kriging with a first-degree detrending (UK1) inverse distance weighting (IDW), and radial basis function (RBF). The number of presences of the species in the study site is also shown.

| Species | Mean AUC | | | | Presences |
| --- | --- | --- | --- | --- | --- |
|  | MBES | UK1 | IDW | RBF |  |
| *A. stellatus* | 0.48 | 0.40 | 0.44 | 0.45 | 99 |
| *G. grandoculis* | 0.57 | 0.58 | 0.56 | 0.64 | 78 |
| *L. sceleratus* | 0.54 | 0.58 | 0.54 | 0.69 | 55 |
| *L. macrorhinus* | 0.53 | 0.69 | 0.53 | 0.57 | 35 |
| *P. multidens* | **0.94** | **0.92** | **0.92** | **0.91** | 36 |
| *P. typus* | **0.87** | **0.84** | **0.84** | **0.84** | 38 |


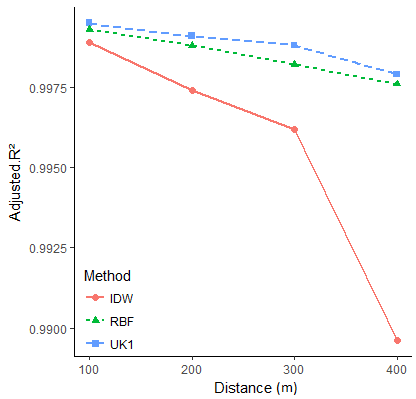


Figure S1. Coefficient of determination (R2) between best SBES the interpolated surfaces and the multibeam data (MBES). Five intervals of distance from the original SBES track are shown for universal kriging with a first-degree of detrending (UK1), inverse distance weighting (IDW), and radial basis function (RBF). In all cases, the linear relationship was significant (p<0.001).


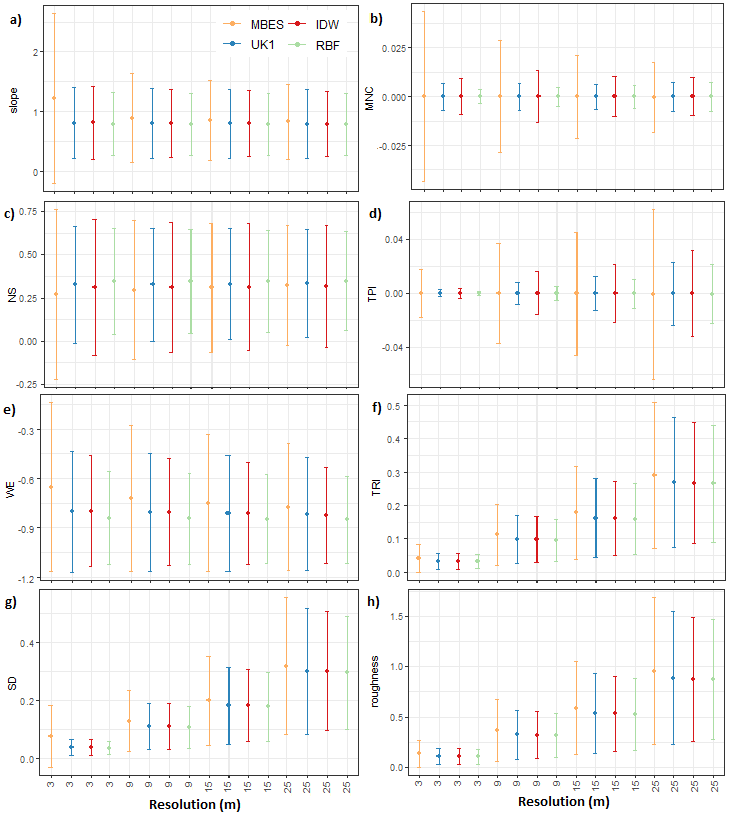


Figure S2. Mean and standard deviation (as error bar) of the depth derivatives based on the MBES and interpolated SBES data using universal kriging with a first-degree detrending (UK1), inverse distance weighting (IDW), and radial basis function (RBF). The four resolutions included in the analysis are shown. a) slope measured in degrees, b) mean curvature (MNC), c) northness (slopes facing north (NS=1), south (NS=-1)), d) topographic position index (TPI), e) eastness (slopes facing east (WE=1), or west (WE=-1)), f) terrain ruggedness index (TRI), g) standard deviation of depth (SD), and h) roughness.


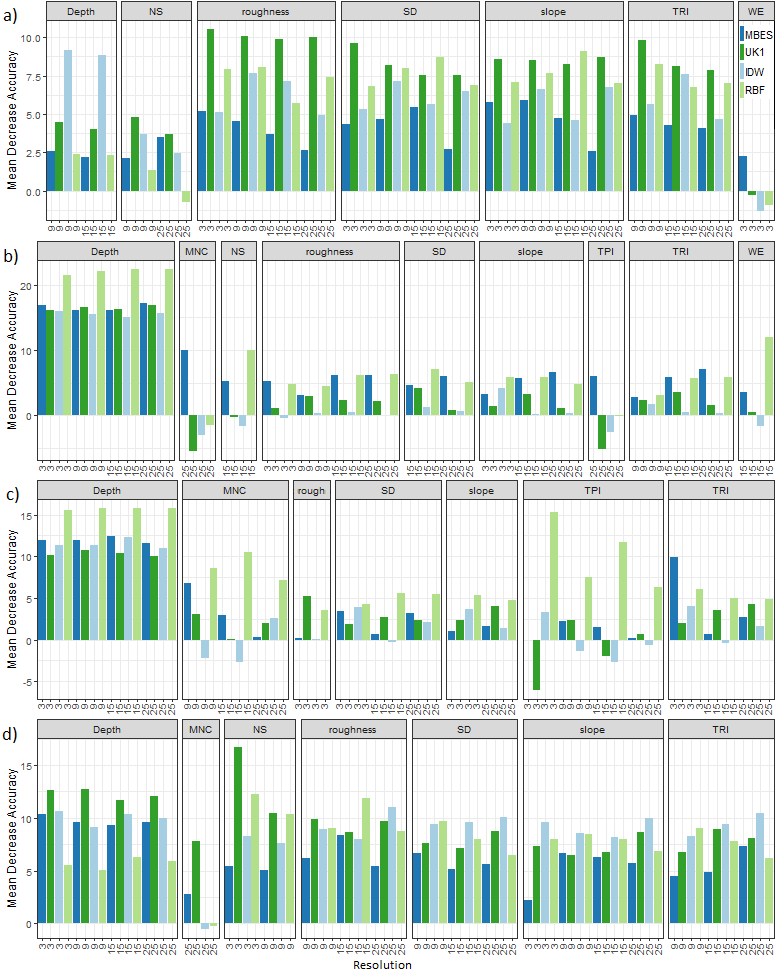


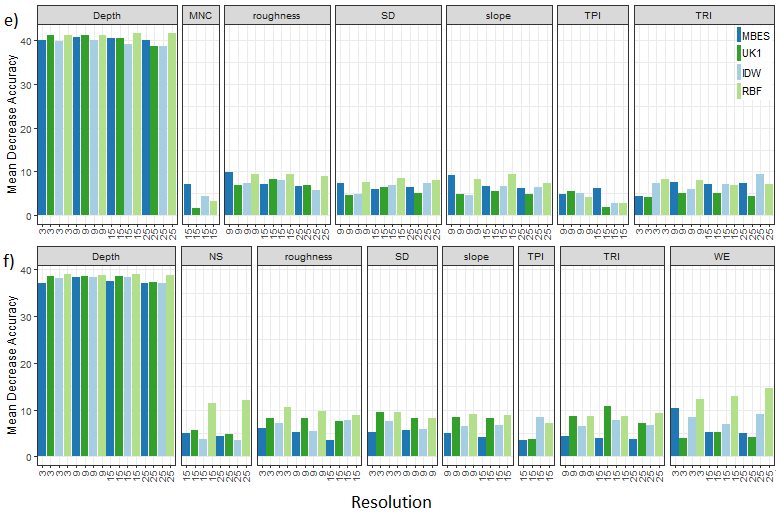


Figure S3. Variables importance in the construction of the Random Forest models using MBES and SBES interpolated data with Universal Kriging with first degree of detrending (UK1), Inverse distance weightening (IDW), and Radial Basis Function (RBF) for a) *A. stellatus*, b) *G. grandoculis,* c) *L. sceleratus,* d) *L. macrorhinus,* e) *P. multidens, and* f) *P. typus*. For brevety, only the 20 most important variables (according to the MBES model) are shown.
